# Evaluating Cross-Sectional Associations Between Cannabis Use and Prospective Memory in People with HIV

**DOI:** 10.1007/s10461-025-04851-3

**Published:** 2025-09-12

**Authors:** Mark K. Britton, Elie Haddad, Yancheng Li, Eric C. Porges, Natalie E. Chichetto, Charurut Somboonwit, Gladys E. Ibañez, Ronald A. Cohen, Robert L. Cook

**Affiliations:** 1 Department of Epidemiology, University of Florida, Gainesville, FL USA; 2 Center for Cognitive Aging and Memory, University of Florida, Gainesville, FL USA; 3 Department of Internal Medicine, University of South Florida, Tampa, FL USA; 4 Department of Epidemiology, Florida International University, Miami, FL USA

**Keywords:** Cognition, Neuropsychology, Cannabis, Substance use, Memory

## Abstract

Prospective memory (PM) deficits are common among people with HIV (PWH) and are linked to poor clinical outcomes. Risk factors for PM deficits in PWH are poorly understood. While cannabis use is associated with worse PM in people without HIV, it is unclear whether this association generalizes to PWH. Three hundred and seven PWH (79% with regular cannabis use) completed the Memory for Intentions Test (MIST). Associations between regular use (vs. no/minimal lifetime use) and MIST score were evaluated. Among participants with regular use, bivariate associations were evaluated between MIST score and self-reported cumulative 30-day THC dose, use frequency, duration of heaviest lifetime use, age of first use, and use motivation (predominantly-recreational, predominantly-therapeutic, or combined). Confounding was addressed with linear regressions adjusted for age and Wechsler Test of Adult Reading. Cannabis use (vs. non-use) was not significantly associated with MIST score in unadjusted or adjusted models (β = − 0.04, 95% CI = − 0.29, 0.21, *p* = 0.74). After confounder adjustment, no associations between cannabis variables and MIST score reached statistical significance. The largest (albeit nonsignificant) effect in adjusted models was found for use motivation: participants with combined use showed better MIST performance vs. predominantly-recreational use (β = 0.28, 95% − 0.02, 0.57, *p* = 0.067). Participants reporting predominantly-therapeutic use vs. predominantly-recreational use performed similarly (β = 0.03, 95% CI = − 0.30, 0.37, *p* = 0.85). PM was not significantly associated with cannabis use in PWH. Associations between motivation for use and PM in PWH warrant further investigation.

**Supplementary Information:**

The online version contains supplementary material available at 10.1007/s10461-025-04851-3.

## Introduction

Prospective memory (PM) is defined as the ability to retain an intended course of action in memory and execute it in an appropriate context. The intention may be retrieved and executed in response to situational cues (event-based PM) or at an appropriate time (time-based PM) [1]. PM is likely subserved partially by strategic monitoring-related areas of the prefrontal cortex; memory-related areas in the medial temporal lobe may also be implicated [2]. Frontostriatal white matter tracts are damaged by HIV [3], and PM deficits are commonly-reported among people with HIV (PWH) [3]. PM deficits are clinically relevant in PWH: lower PM is linked to reduced medication adherence [4–8], reduced ability to perform activities of daily living [9, 10], and reduced health-related quality of life [11] in PWH. Consequently, prevention or reversal of PM deficits among PWH may improve clinical outcomes and quality of life. However, relatively little is known about risk factors for PM deficits in PWH [3].

Among people without HIV (PWoH), chronic cannabis use has been linked to deficits in memory [12, 13], including deficits in PM [14–19]. Cannabinoid receptors are highly concentrated in the hippocampus [20] and are downregulated in the context of regular cannabis use [21], potentially altering memory encoding and retrieval. While the results of individual studies are mixed and several studies have reported no significant association [22–24], a 2016 meta-analysis by Schoeler and colleagues reported a moderate Cohen’s *d* of 0.61 for cannabis-related PM deficits in PWoH [25]. Within PWoH who use cannabis, longer duration of use [17, 26], total lifetime dose [17], and greater average dose [17] have been linked to greater PM deficit, whereas cannabis dependence and product type (i.e., flower vs. concentrate) have not [24, 27]. Findings for use frequency are mixed [17, 18]. However, most studies of cannabis use and PM in PWoH have recruited highly-educated and healthy young adults [14, 17, 23, 24]. Consequently, findings may be confounded by participant characteristics [28] or may not generalize to therapeutic users or to clinical populations.

Cannabis-cognition associations in PWoH may not generalize to PWH. Cannabis use is prevalent among PWH, with 30% of participants in the multisite CFAR Network of Integrated Clinical Systems (CNICS) cohort reporting current cannabis use between 2017 and 2019 [29]; an estimated 25% of PWH in the United States use cannabis therapeutically [30]. Although the nature and causality of cannabis-cognition associations in PWH is disputed [31], cannabis may be neuroprotective in PWH [31, 32] and has been associated in observational research with better cognition [33]. However, to our knowledge, only one study has examined associations between cannabis use (rather than lifetime history of any SUD [3]) and PM performance in PWH. Higher scores on the ASSIST 3.0 cannabis subscale were associated with worse PM in young adult PWH [34]; however, this association was found only on one subscale of a novel PM task and remains to be replicated. Furthermore, PWH who use cannabis are more likely (vs. PWH who do not use cannabis) to report perceived cannabis-related cognitive benefit and less likely to report perceived risk to memory and overall cognition [35]. However, it is unclear whether objective cannabis-PM associations (if any) correspond to self-reported subjective memory benefits in PWH who use cannabis.

The current analysis examined association between several aspects of cannabis use and objective laboratory-based PM performance among a sample of PWH in Florida. Our first aim examined group differences in PM performance between individuals reporting regular past-month cannabis use and individuals reporting no or minimal lifetime cannabis use, controlling for covariates associated with PM in bivariate analyses. We hypothesized that cannabis use would be associated with worse PM. Because time-based PM may be particularly reliant on strategic monitoring and tends to show greater deficits in PWH (vs. event-based PM) [3], we also examined group differences (regular use/no or minimal use) in time- and event-based PM performance; to differentiate PM-specific effects from more general effects on memory, we also examined group differences in retrospective recognition performance. Our second aim examined associations between a range of cannabis use variables (Cannabis Use Disorder (CUD), higher estimated past-month THC consumption, daily vs. nondaily use, longer duration of heaviest period of use, earlier age of first cannabis use, and motivations for cannabis use) and PM performance among those with regular cannabis use. We hypothesized that CUD, heavier current use, greater duration of lifetime heaviest use, and recreational use (vs. therapeutic use) would be linked to worse performance after adjustment for relevant covariates. Our third, exploratory aim was to assess association between objective PM performance and self-reported effect of cannabis on memory and planning among those with regular cannabis use. We hypothesized that perceived negative effects of cannabis on memory and planning would be associated with worse PM and that perceived positive effects would be associated with better PM.

## Methods

### Participants

PWH were recruited from Gainesville, Tampa, and Miami, FL, to participate in an NIH-supported longitudinal study of the health effects of cannabis use among PWH (#R01DA042069) [36]; other findings from this study have previously been reported by our group [37–46]. Participants were recruited at county health departments and community clinics, by flyers in the community, and by word of mouth. Eligibility criteria were the following: 18 years of age or older; confirmed HIV diagnosis; ability to communicate in English; plans to reside in Florida for at least 12 months; and either regular cannabis use (positive cannabinoid urine screen and ≥ 4 self-reported incidents of cannabis use in the past month) or no/limited lifetime cannabis use (negative urine screen, no self-reported use in the past 5 years, and no regular lifetime use). Of 333 participants recruited for the parent study, 307 (92%) participants completed the MIST and were included in this analysis. All data reported were collected at the baseline visit. Data were collected from 2018 to 2021. Study procedures were approved by the Institutional Review Boards of the University of Florida (201702654), Florida International University (201702654-IAA), and the Florida Department of Health (2018-12UF-UF), and conformed to the principles of the Declaration of Helsinki.

### Measures

#### Participant Characteristics

Participants self-reported age, assigned sex, race (Black/non-Black), ethnicity (Hispanic/non-Hispanic), and years of education. Pain during the past 4 weeks was assessed by self-report (yes/no). Daytime sleepiness in the past 30 days (none/less than once a week/once or twice a week/3 or more times a week) was assessed by self-report using the Pittsburgh Sleep Quality Index [47]; “once or twice a week” and “3 or more times a week” were combined for analysis due to infrequent report. Depressive symptoms in the past two weeks were assessed with the PHQ-8 [48]; Cronbach’s α for the PHQ-8 in our sample was 0.87, indicating good internal consistency.

#### Substance Use

Powder and crack cocaine use (yes/no) and cigarette smoking (yes/no) in the past 12 months were assessed by self-report. The AUDIT-10 was used to assess Alcohol Use Disorder symptoms, as well as the frequency and quantity of typical alcohol use [49], in the past 12 months; Cronbach’s α for the AUDIT-10 in our sample was 0.85, indicating good internal consistency. Among participants with cannabis use, the CIDI-Substance Abuse Module [50] was used to assess lifetime DSM-5 Cannabis Use Disorder (yes/no, cut point ≥ 2 symptoms), age of first cannabis use, and duration (years) of heaviest lifetime cannabis use period. Duration of use was log-transformed due to right-skewness approximating a log-normal distribution; transformed data approximated a Gaussian distribution.

The Timeline Follow-Back (TLFB), a retrospective self-report measure of daily substance use over a defined period [51], measured self-reported patterns of cannabis use over the 30 days before the study visit. Total mg of THC over the 30-day period was estimated as the sum of reported mg THC in edibles, vapor, and cannabis concentrates and total reported grams of flower cannabis multiplied by the self-reported estimated percentage of THC; if THC percentage in flower cannabis was not reported, 15% was imputed, approximating the percentage of THC in flower cannabis samples reported by the United States DEA from 2017 to 2021 [52, 53]. Total mg THC was log-transformed due to right-skewness. The distribution of days of use was left-skewed; consequently, this variable was categorized for analysis (daily/nondaily). Our group has previously reported additional information on TLFB variables in this sample [45, 46].

The primary motive for cannabis use (therapeutic or recreational) was self-reported on a sliding scale (0% recreational to 100% recreational). Participants who indicated at least 75% therapeutic or recreational cannabis use were classified as using cannabis predominantly-therapeutically or predominantly-recreationally, respectively. If neither percentage was at least 75%, participants were deemed to have combined motivation for use.

##### Memory for Intentions Test

The Memory for Intentions Test (MIST) [54] is an objective laboratory-based measure of PM. The MIST has been widely used among PWH and has been linked to clinical outcomes, such as HIV medication adherence [5–7]. The clinical scoring procedure was used [55]. Participants complete a distractor task while monitoring a clock and responding to event-based (e.g. “When I hand you a red pen, sign your name on your paper”) and time-based prompts (e.g. “In 15 minutes, tell me that it is time to take a break.”). Event trials were scored 0 (incorrect response) or 2 (correct response to the cue); time trials were scored 0 (incorrect response at incorrect time), 1 (incorrect response at correct time or vice versa), or 2 (correct response at correct time). Time and event subscale scores range from 0 to 8. Total uncorrected summary score ranges from 0 to 48, with higher scores representing better performance. Scores were not demographically-corrected, following prior studies of MIST performance in PWH [5, 56]. The MIST demonstrates satisfactory internal consistency and interrater reliability [57].

After completing the primary task, participants are given a multiple-choice subtest assessing retrospective recognition (e.g., “When I gave you a red pen, were you supposed to 1) sign your name, 2) write your date of birth, or 3) take it home with you?”). Scores on the retrospective recognition subtest range from 0 to 8. Low retrospective recognition scores likely reflect deficits in retrospective memory (RM) encoding; consequently, low MIST scores in the context of low RM may reflect a more general memory deficit rather than deficits in PM per se [58].

##### Wechsler Test of Adult Reading (WTAR)

The Wechsler Test of Adult Reading (WTAR) [59] was used to evaluate potential confounding by group differences in crystallized (i.e., premorbid) cognitive function. In racial/ethnic minorities in the US, the WTAR may outperform years of education as a proxy for crystallized cognitive function [60].

##### Self-Reported Planning and Memory

Participants with cannabis use self-reported the impact of cannabis use (better/worse/no effect) on “memory the next day after using” and “ability to plan things that you need to do” in the past 30 days.

### Statistical Analyses

All statistical analyses were conducted in R 4.2.2 [61], using cobalt for covariate balance assessment [62], psych for Cronbach’s α [63], rstatix for bivariate analyses [64], and gtsummary for reproducible data presentation [65]. Linear regression coefficients were standardized with the parameters package [66]. Missing data was addressed with pairwise deletion; no variable other than cocaine use (11%) had greater than 6% missingness.

Relevant covariates were compared between use groups (use/non-use) using absolute standardized mean differences and absolute proportion differences, with values greater than 0.1 interpreted as evidence of imbalance between groups. These balance statistics are not dependent on sample size and consequently may be preferable to hypothesis tests (e.g., t-tests) for covariate balance assessment [67]. Covariates (age, years of education, WTAR score, past 12-month AUDIT-10 score, past 12-month cocaine use, past 12-month cigarette smoking, PHQ-8 score, pain, and fatigue) were tested for association with MIST score; covariates not significantly associated with MIST score in bivariate analyses were dropped from subsequent models. Education was additionally dropped from subsequent models due to substantial construct overlap with WTAR score and potential multicollinearity.

To address Aim 1, unadjusted MIST summary scores were compared between those with and without cannabis use using an independent samples t-test and multiple linear regression. Age and WTAR score were entered as regression covariates. Cohen’s thresholds were used to interpret effect sizes [68]. As a follow-up exploratory analysis, group (regular cannabis use vs. no/minimal use) differences in event-based PM, time-based PM, and retrospective recognition scores were evaluated using Mann-Whitney U-tests due to the non-normality of subscale distributions. Differences in event-based PM vs. time-based PM performance were evaluated with the sign test.

To address Aim 2, cannabis use variables were tested for association with unadjusted MIST summary scores among those with cannabis use. Lifetime CUD, estimated total grams of THC in the previous 30 days, daily/nondaily use in the previous 30 days, duration of heaviest lifetime use period, age of first cannabis use, and motivation for use were examined. Bivariate associations between cannabis variables and MIST score were evaluated with t-tests, Kruskal-Wallis tests followed by post-hoc Dunn tests, or linear regression, as appropriate. To assess the possibility of confounding of these associations by age and premorbid cognition, separate multiple linear regressions were fit with age and WTAR score as covariates. Associations between MIST subscales and individual cannabis variables were not assessed due to the large number of comparisons and consequently high possibility of Type 1 error.

To address Aim 3, associations between self-reported planning and memory variables and MIST summary score among those with cannabis use were tested using Kruskal-Wallis tests.

## Results

### Participant Characteristics

Of 307 participants, 244 (79%) reported cannabis use. Characteristics of the overall sample, stratified by cannabis use group (regular use/no or minimal use), are reported in Table 1. Relative to nonuse, cannabis use was associated with younger age, less education, male sex, and past 12-month cocaine use and cigarette smoking; additionally, participants with regular cannabis use scored higher on the AUDIT-10 and PHQ-8 and lower on the WTAR compared to participants with non-use. Descriptive statistics for cannabis use variables among participants reporting cannabis use are reported in Table 2.

**Table 1:** Demographic and clinical characteristics of included participants (*N* = 307)

Characteristic	*N*	Overall*N* = 307^a^	Past-Month Cannabis Use*N* = 244^a^	No/Minimal Cannabis Use*N* = 63^a^	SMD^b^
Age	307	49.03 (11.83)	48.05 (11.72)	52.86 (11.54)	**0.41**
Black/African American	307	221 (72%)	180 (74%)	41 (65%)	0.09
Hispanic/Latino	307	41 (13%)	32 (13%)	9 (14%)	0.01
Female	307	140 (46%)	100 (41%)	40 (63%)	**0.22**
Years of Education	307	11.87 (2.23)	11.71 (2.23)	12.51 (2.16)	**0.36**
WTAR Score	303	23.05 (11.11)	22.65 (11.06)	24.60 (11.24)	**0.17**
AUDIT-10 Score	298	4.82 (6.16)	5.57 (6.53)	1.92 (3.06)	**0.72**
Past-Year Smoking	301	188 (62%)	172 (72%)	16 (26%)	**0.46**
Past-Year Cocaine Use	272	86 (32%)	82 (36%)	4 (9.5%)	**0.26**
PHQ-8 Score	291	7.32 (5.88)	7.62 (5.82)	6.15 (6.04)	**0.25**
Pain (past 4 weeks)	303	172 (57%)	139 (58%)	33 (53%)	0.04
Fatigue (past 30 days)	300				
Never		30 (10%)	24 (10%)	6 (9.7%)	< 0.01
Less than once a week		222 (74%)	176 (74%)	46 (74%)	< 0.01
Once a week or more		48 (16%)	38 (16%)	10 (16%)	< 0.01
MIST Score	307	28.88 (11.42)	29.03 (11.27)	28.29 (12.09)	0.06

Standardized mean difference/absolute proportion difference statistics represent covariate imbalance between participants with and without past-month cannabis use; values greater than > 0.10 (bolded) reflect group differences

^a^Mean (SD); n (%)

^b^Absolute standardized mean differences (continuous); absolute proportion differences (binary)

**Table 2:** Cannabis use characteristics among participants with past-month cannabis use (*N* = 244)

Characteristic	*N*	Value^a^
Cannabis Use Disorder	230	106 (46%)
Frequency of Use	239	
Daily		120 (50%)
Non-Daily		119 (50%)
Estimated mg THC/30 Days	236	7,214.04 (10,340.83)
Age of First Use	230	16.06 (4.74)
Duration of Heaviest Use Period (Years)	229	7.05 (9.54)
Motivation for Use	238	
Therapeutic		68 (29%)
Recreational		44 (18%)
Combined		126 (53%)

^a^n (%); Mean (SD)

Age, WTAR score, and years of education were significantly associated with MIST performance, with small-to-medium effect sizes, whereas no significant associations were seen for cocaine use, smoking, AUDIT-10 score, PHQ-8 score, pain, or fatigue (Table 3).

**Table 3:** Bivariate associations of participant characteristics with MIST score

Variable	Pearson *r*	95% CI	*p*
Age	−0.40	−0.49, −0.30	< 0.001
Years of Education	0.21	0.10, 0.32	< 0.001
WTAR	0.36	0.26, 0.45	< 0.001
AUDIT-10 Score	−0.03	−0.14, 0.08	0.63
PHQ-8 Score	−0.004	−0.12, 0.11	0.95

Variable	MIST Score^a^	p	Effect Size^b^
Past-Year Cocaine		0.58	0.07
Yes	28.3 (12.7)		
No	29.2 (11.2)		
Past-Year Smoking		0.97	0.004
Yes	28.9 (11.8)		
No	28.9 (10.8)		
Pain		0.17	0.16
Yes	28.0 (10.8)		
No	29.9 (12.1)		
Fatigue		0.29	0.002
Never	28.8 (11.5)		
Less than once a week	31.8 (8.17)		
Once a week or more	26.9 (12.3)		

^a^Mean (SD)

^b^Cohen’s d; η^2^

### Association Between Any Cannabis Use and Prospective Memory

**Fig. 1:**
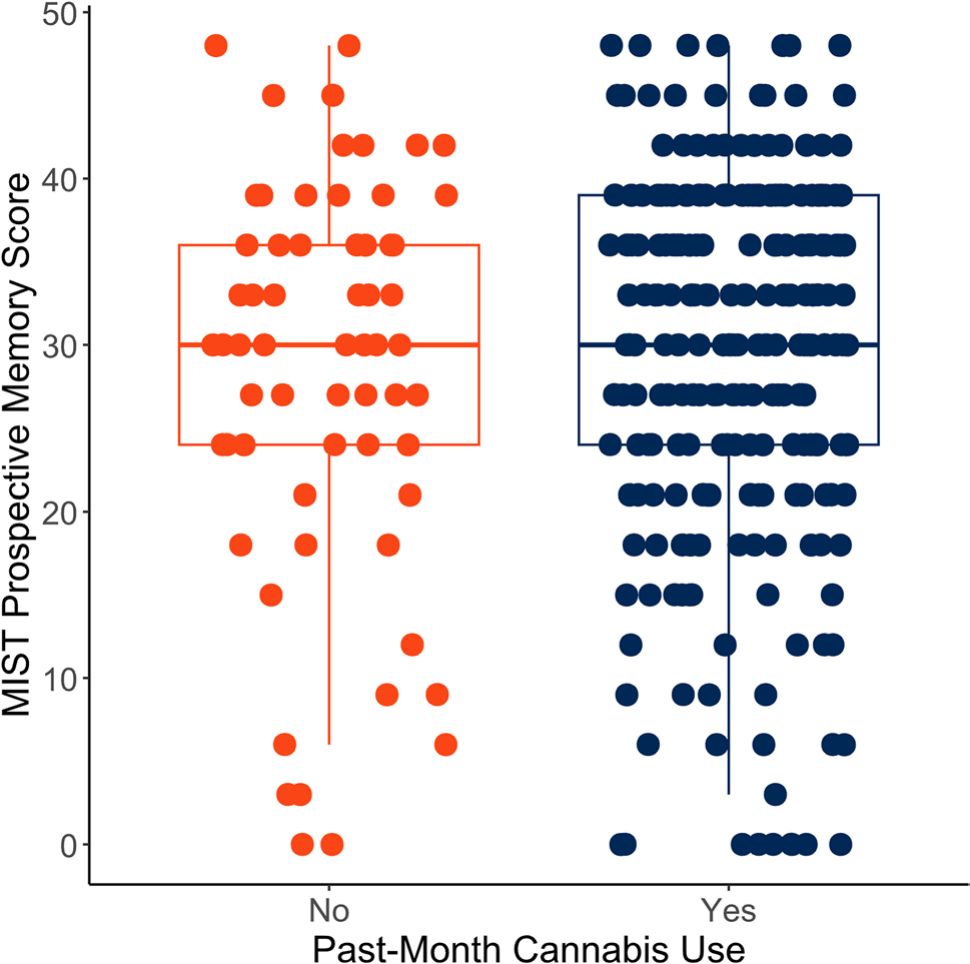
MIST summary score was not significantly associated with past-month cannabis use (vs. no/minimal lifetime cannabis use) (*N* = 307)

In bivariate analyses, we observed no significant MIST differences between participants with cannabis use (mean: 29.03 (11.07)) and participants with no/minimal use (mean: 28.29 (12.09); t(91.7) = −0.44, *p* = 0.66, Cohen’s d < 0.001; Fig. 1). Similarly, no significant association between cannabis use and MIST score was found in linear models adjusted for age and WTAR score (β = −0.04, 95% CI = −0.29, 0.21, *p* = 0.74).

Participants reporting regular cannabis use (median: 4; IQR: 3–5) and no/minimal cannabis use (median: 4; IQR: 3–5) did not differ significantly on time-based PM subscale performance (U = 7216, *p* = 0.45, effect size *r* = 0.04; Supplementary Fig. S1). Similarly, participants with regular cannabis use (median: 4; IQR: 6–8) and no/minimal use (median: 4; IQR: 6–8) did not differ significantly on event-based PM subscale performance (U = 7713, *p* = 0.97, effect size *r* = 0.003; Supplementary Fig. S1). Across the full sample, performance on the event-based subscale was significantly better (vs. the time-based subscale; S(251) = 194, *p* < 0.001). Among the 301 (98%) participants who completed the retrospective recognition MIST subtest, performance did not differ significantly (U = 7515.5, *p* = 0.85, effect size *r* = 0.01; Supplementary Fig. S2) between participants with regular use (median: 7; IQR: 7–8) and no/minimal use (median: 8; IQR: 6–8).

### Association Between Cannabis Use Characteristics and Prospective Memory

#### Cannabis Use Disorder

In bivariate analyses, lifetime CUD (mean = 30.1(10.7)) showed a small effect size for higher MIST score vs. no lifetime CUD; associations were not statistically significant (mean = 27.3(11.4); t(226) = −1.97, *p* = 0.050, Cohen’s *d* = −0.26). In a linear model adjusting for age and WTAR score, CUD was associated with a 0.14 SD increase in MIST score; however, associations did not reach statistical significance (β = 0.14, 95% CI = −0.09, 0.38, *p* = 0.22).

#### Dose

Greater log-transformed mg of THC in the past 30 days was not associated with MIST score in unadjusted (β = 0.11, 95% CI = −0.02, 0.24, *p* = 0.097) or adjusted models (β = 0.03, 95% CI = −0.09, 0.14, *p* = 0.62).

#### Frequency of Use

In bivariate analyses, frequency of use was not significantly associated with MIST score, with a negligible effect size (t(237) = −1.12, *p* = 0.27, Cohen’s d = −0.14). Participants with daily use had a mean score of 28.2 (SD = 11.3), whereas participants with non-daily use had a mean of 29.9 (SD = 11.2). In adjusted models, participants with non-daily use performed 0.15 standard deviations better on the MIST compared to participants with daily use; associations were not statistically significant (β = 0.15, 95% CI = −0.07, 0.37, *p* = 0.17).

#### Duration of Heaviest Lifetime Use Period

Greater duration of heaviest lifetime use period (years) was significantly associated with lower MIST score (β = −0.13, 95% CI = −0.26, −0.001, *p* = 0.048). However, this association was attenuated in adjusted models (β = −0.09, 95% CI = −0.20, 0.03, *p* = 0.15).

#### Age of First Use

Age of first use was not significantly associated with MIST score in unadjusted (β = −0.07, 95% CI = −0.20, 0.06, *p* = 0.30) or in adjusted models (β = −0.06, 95% CI = −0.17, 0.06, *p* = 0.32).

#### Motivation for Use

18% of participants who used cannabis reported primarily recreational use. 29% reported primarily therapeutic use. 53% reported combined recreational-therapeutic use.

**Fig. 2:**
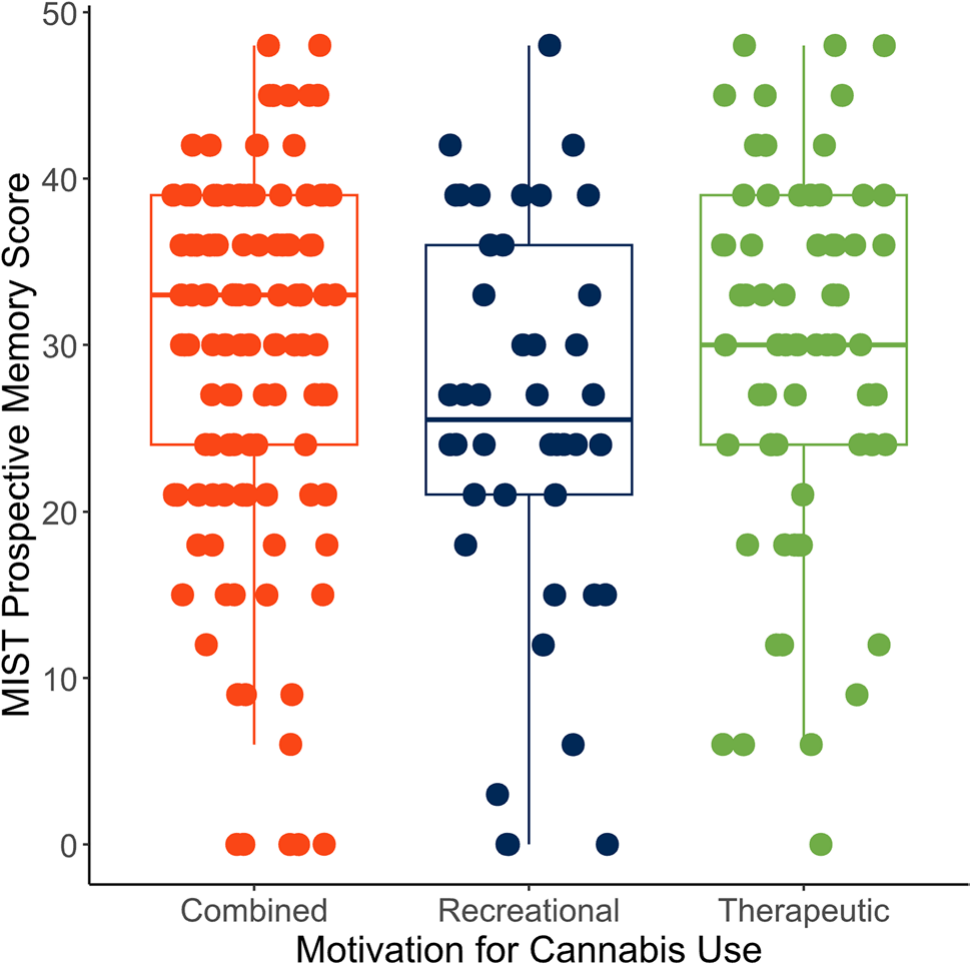
MIST summary scores were lower in bivariate analyses in participants with predominantly-recreational use; however, group differences did not reach significance (*N* = 238)

In bivariate analyses, motivation for use was not significantly associated with MIST score overall, with a small effect size (H(2) = 4.76, *p* = 0.09, effect size η^2^ = 0.012; Fig. 2). The mean MIST score in participants with combined recreational-therapeutic use was 29.8 (SD: 10.9). Mean performance in participants with predominantly-recreational use was 25.6 (SD: 12.0). Participants with predominantly-therapeutic use had a mean of 29.6 (SD: 11.2).

In analyses adjusted for age and WTAR score, participants with combined recreational-therapeutic use performed 0.28 standard deviations better than participants with predominantly-recreational use (β = 0.28, 95% −0.02, 0.57, *p* = 0.067). Participants with predominantly-therapeutic use performed 0.03 standard deviations better than participants with predominantly-recreational use in age- and WTAR-adjusted analyses (β = 0.03, 95% CI = −0.30, 0.37, *p* = 0.85). Associations were not statistically significant.

### Association Between Objective PM and Self-Reported Memory and Planning in PWH with Cannabis Use

Two hundred and twenty-five participants with past-month cannabis use self-reported the impact of cannabis use on planning and memory. Of this subset, 83 (37%) reported improved planning, 21 (9%) reported worsened planning, and 121 (54%) reported no change. Fifty-nine (26%) reported improved memory, 18 (8%) reported worsened memory, and 148 (66%) reported no change.

There was no statistically significant association between MIST score and self-reported effect of cannabis on planning (H(2) = 0.54, *p* = 0.76, η^2^ = −0.006) or memory (H(2) = 1.76, *p* = 0.41, η^2^ = −0.001). Effect sizes η^2^ were small. Full results are reported in Table 4.

**Table 4:** Self-reported effect of cannabis on memory and planning was not associated with objective MIST score (*N* = 225)

Variable	MIST Score^a^	*p*	Effect Size η^2^
Effect of Cannabis on Memory		0.41	−0.001
Better	27.6 (11.4)		
Worse	32.0 (10.4)		
No Difference	29.4 (11.2)		
Effect of Cannabis on Planning		0.76	−0.006
Better	27.6 (12.1)		
Worse	29.1 (11.2)		
No Difference	29.7 (10.5)		

^a^Mean (SD)

## Discussion

Although cannabis use has been linked to deficits in PM in PWoH [25], reported findings are inconsistent and the generalizability of findings to PWH is under-explored. Furthermore, it is not known which aspects of cannabis use (if any) are most relevant to PM and to what extent associations may be confounded by other participant characteristics (e.g., age and crystallized cognition). The present study examined associations between PM and a range of cannabis use variables in a large cross-sectional sample of adults with HIV.

We observed no association between regular cannabis use (relative to no/minimal lifetime use) and overall PM in either bivariate analyses or analyses adjusted for age and crystallized cognition; nor was cannabis use associated with performance on either event-based PM or time-based PM subscales. Effect sizes for bivariate analyses were negligible. This finding stands in contrast to prior reports that cannabis use is cross-sectionally linked to PM in PWoH [25], suggesting that cannabis-PM associations in PWoH may not generalize to PWH. Furthermore, retrospective recognition was similarly not associated with cannabis use (vs. nonuse) in this sample; we have previously reported no association between cannabis use per se and performance on NIH Toolbox Cognition Battery executive and memory measures in our cohort [39]. In other words, we observed no evidence that cannabis is consistently associated with worse or better cognition in PWH. Instead, we observed low PM performance across participant groups. Consistent with prior reports of lower MIST performance in PWH [3], we observed a mean MIST score approximately three standard deviations below published means for healthy adults using the clinical scoring procedure [69].

While we were unable to directly assess causal pathways underlying overall PM deficits in our sample, prior evidence suggests several possible mechanisms. Because HIV damages frontostriatal white matter tracts implicated in strategic monitoring (a critical component of PM) [3, 70], PWH may be less able to detect and appropriately respond to retrieval cues. Consistent with this model and with prior findings in other samples [3], our sample showed lower time-based PM performance than event-based PM performance, consistent with poor strategic monitoring. PM deficits in PWH may also to some extent reflect other neurobiological alterations in PWH, such as dysregulation of executive function or memory encoding and retrieval due to abnormalities in endocannabinoid receptor function [31, 32]. Because cannabis use may normalize endocannabinoid receptor-mediated cognitive deficits (particularly executive deficits) in PWH, along with other physiological benefits (e.g., reduction of inflammation) [31], it is therefore possible that our null finding for cannabis use reflects a combination of beneficial and detrimental effects of cannabis in PWH. However, this mechanism has yet to be directly demonstrated and therefore remains speculative.

The observed PM deficit may also reflect non-HIV sample characteristics, such as age, educational attainment, or crystallized cognition, all of which are associated with PM [54]. Our sample was older and less well-educated than typical participants in prior studies of cannabis use and objective PM, the majority of which sampled young adults (including university students) [14, 17, 19, 23, 24]. However, even when adjusting models for age and crystallized cognition, we did not replicate cannabis-PM associations previously reported in PWoH. Overall, then, our findings are consistent with a general HIV effect on PM.

Within the subset of participants reporting recent cannabis use, the largest effect size after adjustment for potential confounders was seen for motivation for use. In models adjusted for age and crystallized cognition, participants with combined recreational-therapeutic motivation for use showed a 0.28 SD increase in PM relative to primarily-recreational use; however, within-group heterogeneity was substantial and this association was not significant at conventional thresholds, suggesting that this observation should be interpreted with caution. Unexpectedly, predominantly-recreational and predominantly-therapeutic use groups differed by only 0.03 SD in adjusted models. The clinical significance of these findings (if any) is unclear.

We observed little evidence for meaningful PM differences associated with other cannabis variables; although associations were apparent in bivariate models for THC dose, lifetime CUD (vs. no CUD), and duration of heavy use, these associations were substantively attenuated after adjustment for age and crystallized cognition. No associations were apparent between frequency of use or age of first use and PM. Collectively, these null findings suggest that greater lifetime exposure to cannabis is not linked to PM in PWH. Consequently, PM differences associated with use motivation are likely not explained by differences in cumulative cannabis exposure per se. Rather, motivation-PM associations may result from differences in preferred cannabis strain or pattern of use [71, 72] or residual confounding by other participant characteristics.

Despite little evidence for cannabis-PM associations in our sample, participants commonly reported subjective cognitive benefits from cannabis use. 26% of participants with past-month cannabis use self-reported beneficial effects of cannabis use on memory, and 37% self-reported beneficial effects on planning. However, we observed no association between self-reported impact of cannabis on memory and planning and objective PM performance. Disconnects between objective and subjective PM performance have previously been reported in PWoH who use cannabis [14, 23], although the direction of associations is inconsistent. Discrepancies between objective and subjective PM performance have also been reported in PWH [73], potentially due to overlapping neural substrates underlying prospective memory and metamemory (awareness of own memory performance). Our findings therefore provide preliminary evidence of metamemory deficiencies among PWH who use cannabis. Deficits in self-awareness and self-monitoring may alter decision-making around the risks and benefits of cannabis use [74] and have been implicated in substance use disorders [75]. However, the real-world implications of metamemory deficits in PWH who use cannabis are not known, and further research is needed in this area.

To our knowledge, the present analysis is the first comprehensive examination of associations between features of cannabis use and PM in a large sample of PWH. However, our study was subject to several limitations. First and most importantly, we were unable to compare cannabis-PM associations in PWH and PWoH, as our sample did not include PWoH. Consequently, although a HIV effect on PM has been reported frequently in other samples, we were unable to evaluate HIV-related differences directly in our sample. Second, cannabis and other substance use were assessed by self-report, potentially leading to misclassification (although recent cannabis use was confirmed with urine biomarkers) or to underreporting of substance use. Missingness for past-year cocaine use was particularly high, which may represent nonresponse in individuals with past-year use (i.e., missingness not at random) and may have attenuated associations with cocaine use. Similarly, we did not directly test associations between cannabis strain and PM performance, although motivation for use may be associated with cannabis strain preference (e.g., preference for high-THC or high-CBD strains). Fourth, we did not exclude participants for recent cannabis use; consequently, some individuals’ performance may have been affected by recent acute use. Fifth, we examined multiple aspects of cannabis use, which increases opportunity for Type 1 error. There is currently no consensus on which aspects of cannabis use are most relevant to cognitive performance in PWH; future research might benefit from a standardized cannabis exposure variable incorporating multiple aspects of use. Finally, although the MIST is a validated laboratory measure of prospective memory, it may not fully capture all aspects of real-world planning and prospective memory.

In sum, the current analysis does not support the hypothesis that cannabis use is directly detrimental to PM among PWH. Discontinuation of cannabis use therefore may not improve PM performance and downstream clinical outcomes in PWH who use cannabis, although this remains to be tested empirically. However, we observed an overall PM deficit in our sample. Additionally, we observed preliminary evidence for deficits in self-evaluation of objective memory performance, which may potentially impact subjective perceptions of cannabis risks and benefits. Overall, our findings highlight PM as an area of concern in PWH and underline the need for further research on risk factors for PM deficit in this population.

## Supplementary Information

Below is the link to the electronic supplementary material.

Supplementary material 1 (DOCX 608.5 kb)

## Data Availability

Data are available from the Southern HIV and Alcohol Research Consortium upon reasonable request.

## Code availability

Code is available from the Open Science Framework at https://osf.io/5uz23/.
