# Supplementary material for: Evaluating Cross-Sectional Associations Between Cannabis Use and Prospective Memory in People with HIV
Source: AIDS Behav. 2025 Sep 12;30(1):107–17. doi: 10.1007/s10461-025-04851-3 (PMC12815984; doi:10.1007/s10461-025-04851-3)

**Supplementary Fig. 1** Past-month cannabis use was not associated with event-based prospective memory or time-based prospective memory subscale performance in PWH (N = 307)


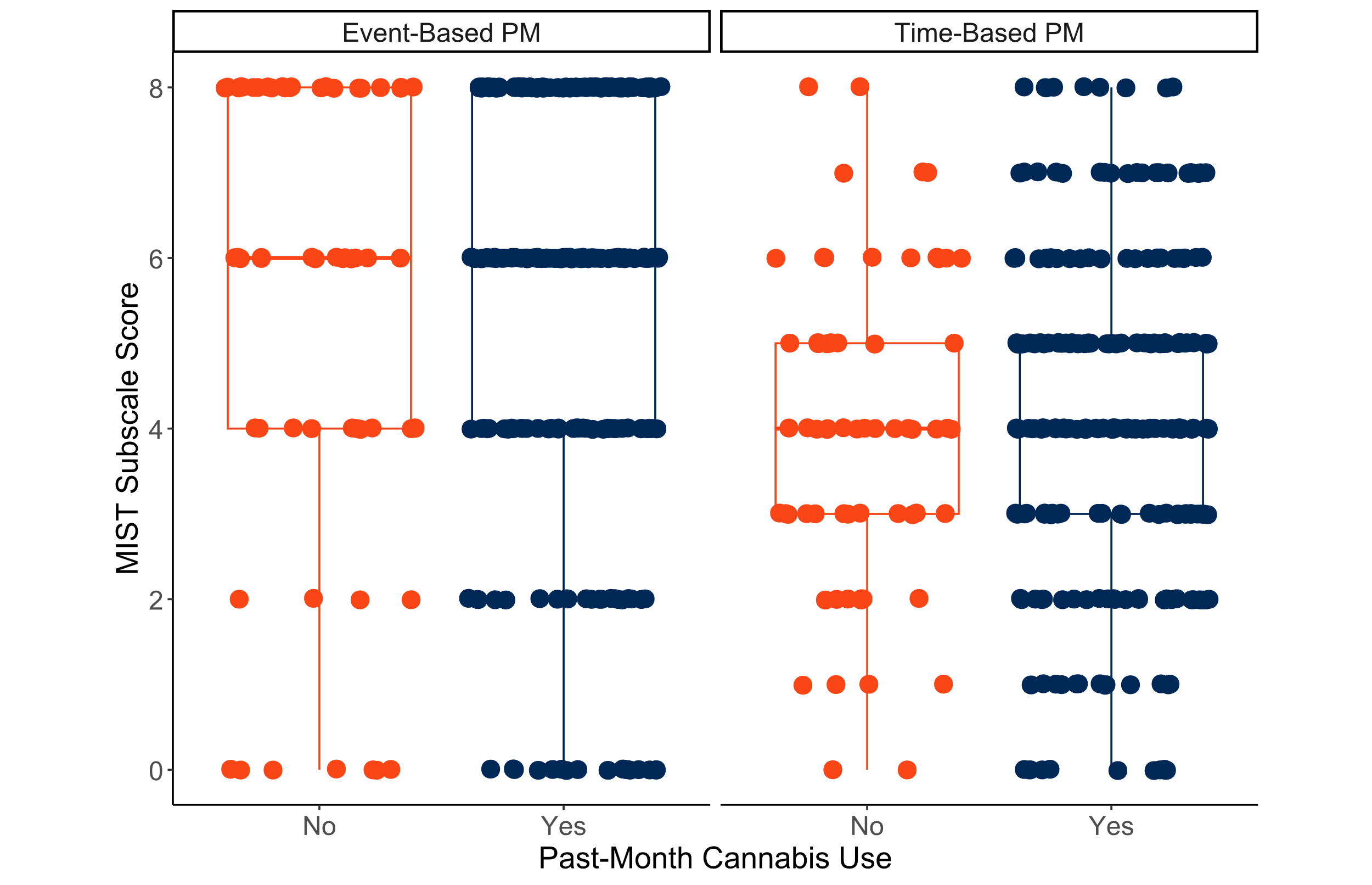


**Supplementary Fig. 2** Past-month cannabis use was not associated with retrospective recognition performance in PWH (N = 301)


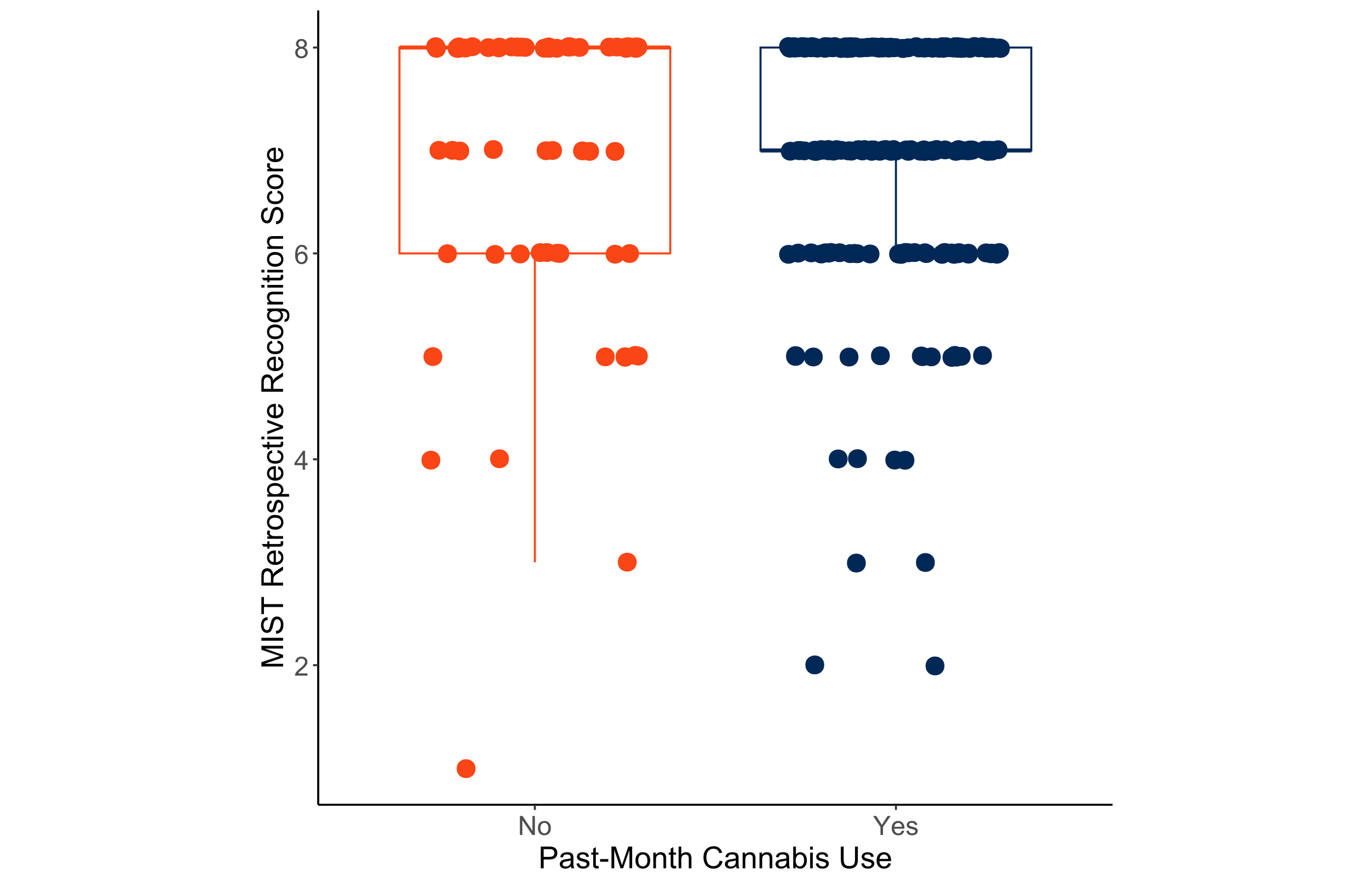

Supplement: Supplementary file 1 — Supplementary material 1 (DOCX 608.5 kb) [file 10461_2025_4851_MOESM1_ESM.docx]
